# Clinical Features of Urticaria: Results From a Hospital-Based Multicenter Study in China

**DOI:** 10.3389/fmed.2022.899857

**Published:** 2022-06-09

**Authors:** Xin Wang, Li-Juan Liu, Lin-feng Li, Xiao-Dong Shi, Yi-Wei Shen

**Affiliations:** ^1^Department of Dermatology, Beijing Friendship Hospital, Capital Medical University, Beijing, China; ^2^Department of Dermatology, The First Hospital of Hebei Medical University, Shijiazhuang, China; ^3^AI Research and Development Center, China Telecom Research Institute, Beijing, China; ^4^Department of Dermatology, Beijing Shijitan Hospital, Capital Medical University, Beijing, China

**Keywords:** dermato-epidemiology, epidemiological characteristics, multicenter, chronic spontaneous urticaria, H1-receptor antagonists

## Abstract

**Background:**

The clinical features of urticaria have not been fully illustrated.

**Objectives:**

To demonstrate clinical features of urticaria in different areas of southern and northern China.

**Methods:**

In this hospital-based multicenter study, outpatients with urticaria filled in a questionnaire during the initial visit and follow-up (once per week, lasting for a month).

**Results:**

Overall, 1,715 outpatients with urticaria with a mean age of 37.86 ± 16.08 years (range = 0.5–87 years) were recruited. The median disease duration was 1.94 ± 4.31 years (range = 0–58 years). More itching was observed in the northern areas higher than that in the southern areas (99.5 vs 94.1%, *P* < 0.001). The incidence of pain, arthralgia, and family history in southern areas was higher than that in northern areas (5.1 vs 1.1%, 9.6 vs 0, 10.6% vs 3.2%, *P* < 0.001). The leading subtypes of specified urticaria were chronic spontaneous urticaria (81.4%) and symptomatic dermographism (35.9%). The incidence of symptomatic dermographism and cold urticaria in the southern areas was lower than that in the northern areas (31.8 vs. 50.3%, 4 vs. 8.5%, *P* < 0.001). Allergic diseases were the most common concomitant disorders of urticaria. More than half of the patients had to avoid certain food, such as fish-prawn-crab (30.7%) and alcohol (20%). Ebastine (41.1%) was the most commonly prescribed drug. The disease duration negatively correlated with the severity of itching and number of wheals (>50/24H) (Spearman’s rank correlation test, *p* < 0.001).

**Conclusion:**

This study provides a profile of clinical characteristics of urticaria in China and filled the gap in the field of regional comparative studies on urticaria.

## Introduction

Urticaria is a common, mast cell-driven disease, presenting symptoms like wheals, angioedema, or both. The lifetime prevalence of acute urticaria (AU) is approximately 20% (1). Based on a large database from an American commercial insurance company, the 1-year period prevalence of chronic urticaria (CU) was 0.08% (2). In European countries, the 1-year period prevalence of CU ranges from 0.38 to 0.8% (3, 4). A cross-sectional study reported that the prevalence of chronic spontaneous urticaria is 2.7% among adolescents in China (5). Generally, CU develops among 20–45% of individuals diagnosed with AU (3). Urticaria, particularly in chronic forms, has a negative impact on the quality of life and causes high costs on health care. Urticaria-related costs may be as high as US $1750 to US $2050 per patient per year (2, 6).

Urticaria can be classified according to duration and etiology although ≥2 types of urticaria can coexist in the same patient. Different types and subtypes of urticaria are characterized by unique clinical features. Among patients with inducible urticaria in a tropical country, the most common type is symptomatic dermographism (40–73%), while solar urticaria, heat urticaria, and vibratory angioedema are rarer (7–9). At present, very few literature have focused on the classification and distributed features of urticaria in China, so we performed a hospital-based multicenter epidemiologic survey on Chinese outpatients with urticaria. In the present study, we focused on classification and therapy in outpatients with urticaria based on clinical reality.

## Materials and Methods

### Data Source and Subjects

This prospective multicenter study was performed from 1 January 2019 to 31 December 2019. We approached 1,800 outpatients diagnosed with urticaria, among whom 1,715 (95.3%) agreed to participate in this study. All subjects were recruited from 12 tertiary hospitals in six provinces and municipalities in mainland China, including Fujian, Sichuan, Chongqing, Hubei, and Liaoning provinces, as well as Beijing, which are distributed geographically uniform in China. This study was approved by the Institutional Review Board (IRB) Committee of Beijing Friendship Hospital, Capital Medical University, China. The inclusion criterion was as follow: patients diagnosed with urticaria with no specific physical problems that might interfere with the conduct of the interview. Also, the exclusion criteria were patients with communication inability, mental retardation, dementia, severe symptoms of acute psychosis, and severe restlessness. All participants had been selected after providing orally informed consent.

### Study Design and Research Content

As a multicenter study, dermatologists involved in this study were trained in a standardized manner, and they were performed in accordance with relevant guidelines and regulations. No matter what their race, gender, age, and education are, in our study, all participants must comply with the European Academy of Allergology and Clinical Immunology, the Global Allergy and Asthma European Network, World Allergy Organization (EAACI/GA2LEN/EDF/WAO) guidelines (1), or the Version of Chinese Guidelines for the Diagnosis and Treatment of Urticaria. The presence of urticaria was identified on physician-certified diagnosis using the International Classification of Diseases, 10th Revision (ICD-10) codes, including various types of urticaria (L500–L509) and angioedema (T783). Multiple diagnoses were allowed. “Clinical urticaria” is defined as a type of urticaria that makes a patient to visit a hospital and be diagnosed with the main problem by a clinician, including acute urticaria. Specific types of urticaria included chronic spontaneous urticaria (CSU), symptomatic dermographism, delayed pressure urticaria, cold urticaria, heat urticaria, solar urticaria, vibratory angioedema, and aquagenic urticaria. Appropriate diagnostic tests have been performed depending on the nature of the urticaria subtype, for example, the elicit dermographism and threshold test for symptomatic dermographism, the pressure test and the threshold test for delayed pressure urticaria, and the temperature test for heat/cold urticaria (1). In addition to general demographic characteristics, clinical symptoms and signs were explained in detail, with a particular focus on the current and former therapeutic schedule and test results. Ffood items to be avoided and medical history were recorded. Then, patients were examined during follow-up visits once per week for a period of 1 month. All enrolled follow-up patients completed a specific survey on the severity of itching and number of wheals. The urticaria activity score 7 (UAS7) was used to determine the effect of treatment.

### Data Processing and Statistical Methods

All data processing and statistical analyses were performed using Statistical Package for the Social Sciences (version 20.0, SPSS Inc., Chicago, IL, United States). Continuous variables were presented as the median (M) and interquartile range (Q) according to the data distribution. Normality was evaluated by the Kolmogorov–Smirnov test. Statistically significant differences between the groups were determined by the *t*-test or Mann–Whitney *U*-test. For enumeration variables, the rate (%) within the group was reported, and the Pearson chi-square test (two test) was used for the comparison between groups. Missing data were excluded from statistical analysis. A value of *p* < 0.05 was considered statistically significant.

### Patients (Participants) and Public Involvement

There was no specific patient or public involvement in the development of the research questions, outcome measures, study design, and recruitment/conduct of the present study. Science education in regional language helps clinicians to educate patients with urticaria. In addition, to study the participants, the key findings of the study will also be disseminated to the public through science popularization education.

## Results

### Respondent Characteristics (*N* = 1,715)

Twelve hospitals participated in this epidemiological survey and 1,715 outpatients with urticaria were recruited, including patients with acute urticaria (198 cases) and chronic urticaria (1,517 cases). Subjects with urticaria included 43.4% men (744/1,715), with a mean age of 37.86 ± 16.08 years (range = 0.5–87 years). The recruited outpatients were divided into four age groups (<20, 20–40, 40–60, and >60). Except for group <20, all other groups had female predominance, and the difference was statistically significant (*p* < 0.05). The median disease duration was 1.94 ± 4.31 years (range = 0–58 years). Among them, 38 patients (2.2%) were pregnant and 35 patients (2%) were breastfeeding. Of the selected outpatients, 95.8% of patients were diagnosed with pruritus, which was a more common disease than arthralgia (7.5%) and fever (1.3%). The results are shown in Tables 1, 2.

**TABLE 1:** Age and gender composition of survey subjects.

Age group	Women (*n*,%)	Men (*n*,%)	W/M	Total (*n*,%)
<20	79 (8.1)	88 (10.8)	0.898	167 (9.7)
20–40	459 (47.3)	365 (49.1)	1.258	824 (48.1)
40–60	322 (33.2)	215 (28.9)	1.498	537 (31.3)
>60	111 (11.4)	76 (10.2)	1.461	187 (10.9)
Total	971 (100)	744 (100)	1.304	1,715 (100)

*X^2^ = 9.194, p-value = 0.027, Pearson chi-square test.*

**TABLE 2:** Clinical characteristics of each type of urticaria in different regions.

Characteristics	Total *N* (%)	Southern patients *N* (%)	Northern patients *N* (%)	Z/X^2^	*p*-Value
No. of patients	1,715 (100)	1,337 (78)	378 (22)	–	–
**Clinical symptom**					
Mean age at the first clinic visit (years) [M (Q)]	35.47 (24)	35.33 (24)	35.99 (25)	0.149	0.484^a^
Duration of CU before the first clinic visit (years) [M (Q)]	0.53 (0.25)	0.55 (0.25)	0.49 (0.17)	–	0.014^b^*
Itch	1,634 (95.3)	1,258 (94.1)	376 (99.5)	18.952	0.000^c^*
Pain	72 (4.2)	68 (5.1)	4 (1.1)	11.887	0.001^c^*
Arthralgia	129 (7.5)	129 (9.6)	0 (0)	39.438	0.000^c^*
Fever	22 (1.3)	19 (1.4)	3 (0.8)	0.916	0.338^c^
**Diseases/family/drug allergy/history**					
History of allergic rhinoconjunctivitis	323 (18.8)	243 (18.2)	80 (21.2)	1.722	0.189^c^
History of atopic dermatitis	239 (13.9)	161 (12)	78 (20.6)	18.142	0.000^c^*
History of asthma	45 (2.6)	36 (2.7)	9 (2.4)	0.112	0.738^c^
History of thyroid disease	21 (1.2)	8 (0.7)	15 (3.2)	16.18	0.000^c^*
Family history	154 (9.0)	142 (10.6)	12 (3.2)	19.991	0.000^c^*
Drug allergy history	46 (2.7)	34 (2.5)	12 (3.2)	0.450	0.502^c^
**Laboratory inspection**					
Elevated total IgE in serum	146 (8.5)	62 (4.6)	84 (22.2)	117.002	0.000^c^*
Thyroperoxydase (TPO)/thyroglobuline (Tg) positive	160 (9.3)	76 (5.7)	84 (22.2)	95.279	0.000^c^*
Autoantibody positive	126 (7.3)	84 (6.3)	42 (11.1)	10.092	0.001^c^*
H. pylori infection	74 (4.3)	47 (3.5)	27 (7.1)	9.392	0.002^c^*
**Related diseases**					
Chronic spontaneous urticaria	1,396 (81.4)	1,078 (80.6)	318 (84.1)	2.382	0.123^c^
Symptomatic dermographism	615 (35.9)	425 (31.8)	190 (50.3)	43.740	0.000^c^*
Angioedema	163 (9.5)	138 (10.3)	25 (6.6)	4.710	0.030^c^*
Delayed pressure urticaria	88 (5.1)	87 (6.5)	1 (0.3)	23.591	0.000^c^*
Cold urticaria	86 (5.0)	54 (4.0)	32 (8.5)	12.124	0.000^c^*
Heat urticaria	101 (5.9)	93 (7.0)	8 (2.1)	12.453	0.000^c^*
Solar urticaria	32 (1.9)	32 (2.4)	0 (0.0)	9.219	0.002^c^*
Vibratory angioedema	10 (0.6)	9 (0.7)	1 (0.3)	0.849	0.357^c^
Aquagenic urticaria	5 (0.3)	0 (0.4)	5 (0)	1.418	0.234^c^

*Patients from southern areas came from nine hospitals in southern China, including Sichuan, Hubei, and Fujian; patients from northern areas came from three hospitals in northern China including Beijing and Liaoning Province. 1,715 outpatients with urticaria were recruited, including acute urticaria (198 cases) and chronic urticaria (1,517 cases).*

*p < 0.05.

*^a^T-test.*

*^b^Mann–Whitney U-test.*

*^c^Chi-square test.*

### Classification and Proportion of Each Type of Urticaria

The leading subtype of specified urticaria was chronic spontaneous urticaria (1,396 cases, 81.4%). Among them, 422 outpatients were diagnosed with chronic spontaneous urticaria alone, 437 outpatients with chronic spontaneous urticaria combined with symptomatic dermographism, and 138 outpatients with chronic spontaneous urticaria combined with angioedema. Symptomatic dermographism (615 cases, 35.9%) and heat contact urticaria (101 cases, 5.9%) were ranked as the second and the third common types of the disease, respectively. Additionally, three or more different subtypes of urticaria coexisted in 194 outpatients. The friction test that proved positive was seen in 61.0% (990/1,622) of patients with urticaria (missing in 93 cases). The survey also showed that the proportion of symptomatic dermographism and cold urticaria in the northern areas was higher than that in the southern areas (50.3 vs. 31.8%, 8.5 vs. 4%, *p* < 0.001). The clinical characteristics of each type of urticaria in different regions were summarized in Table 2.

### Laboratory Inspection and Comorbid Conditions

In this visit, the proportion of patients with abnormal findings was as follows: the percentage of positive elevated total IgE, TPO/Tg positive, autoantibody-positive, *H. pylori* infection in the northern areas was significantly higher than that in the southern areas (22.2 vs 4.6%, 22.2 vs 5.7%, 11.1 vs 6.3%, and 7.1 vs 3.5%, respectively; all *p* < 0.05, chi-square test). About 9% of patients (154 cases) had a family history of urticaria, and there was a big gap between the southern and the northern areas (10.6 vs 3.2%, *p* < 0.001). Allergic diseases were the most common concomitant disorders of urticaria, such as allergic rhinitis (18.8%) and eczema/dermatitis (13.9%). The proportion of hypertension (5.7%) was relatively higher than diabetes (1.5%) or tumor (1.2%) (Table 2).

### Food Items to Be Avoided

Half of the patients with urticaria had to avoid certain food items; however, only 16.4% of them considered this approach effective. The most popular food items to be avoided by patients with urticaria were fish–prawn–crab (30.7%) and alcohol consumption (20%), followed by beef and mutton (18.6%), milk (9.5%), pepper (8.6%), egg (6.3%), nuts (1.8%), and beans (1%).

### Treatment Options

The second-generation H1-antihistamines were the most commonly prescribed drugs, such as Ebastine (41.1%), levocetirizine (36.2%), and olopatadine (16%). There was no significant difference between acute and chronic urticaria in patients prescribed with second-generation H1-antihistamines (92.1 vs 88.4%, *p* > 0.05), and the proportion of patients with acute urticaria was significantly higher than patients with chronic urticaria concerning first-generation H1-antihistamines (22.7 vs 10.5%, *p* < 0.001). As a non-antihistamine compound, glycyrrhizin (34.3%) was the most widely used drug, followed by traditional Chinese medicine (9.2%) and Tripterygium glycoside (8.1%). In terms of treatment options, acrivastine and hydroxychloroquine were more commonly used in patients with a disease course longer than 1 year, which is higher than that in patients with a shorter disease course, while patients with a disease course shorter than 1 year have more prescriptions for compound glycyrrhizin and olopatadine than those patients with a longer disease course; both the differences are statistically significant (*p* < 0.05). Therapeutic options in this visit were summarized in Table 3.

**TABLE 3:** Therapeutic options in outpatients with urticaria in different course by clinical reality.

Medicine	Total (%)	≤1 year (%)	>1 year (%)	X^2^	*p*-Value
Ebastine	705 (41.1)	519 (41.9)	186 (39.1)	1.124	0.289
Levocetirizine	620 (36.2)	454 (36.6)	166 (34.9)	0.466	0.495
Compound glycyrrhizin	589 (34.3)	452 (36.5)	137 (28.8)	9.041	0.003*
Olopatadine	274 (16)	219 (17.7)	55 (11.6)	9.598	0.002*
Loratadine	234 (13.6)	160 (12.9)	74 (15.5)	2.023	0.155
Cetirizine	221 (12.9)	151 (12.2)	70 (14.7)	1.943	0.163
Fexofenadine	193 (11.3)	143 (11.5)	50 (10.5)	0.371	0.543
Traditional Chinese medicine	158 (9.2)	122 (9.8)	36 (7.6)	2.144	0.143
Tripterygium glycoside	139 (8.1)	95 (7.1)	44 (9.2)	1.147	0.284
Ketotifen	124 (7.2)	94 (7.6)	30 (6.3)	0.846	0.358
Acrivastine	94 (5.5)	59 (4.8)	35 (7.4)	4.456	0.035*
Desloratadine	86 (5)	65 (5.2)	21 (4.4)	0.503	0.478
Hydroxychloroquine	39 (2.3)	18 (1.5)	21 (4.4)	13.548	0.000*
Chlorphenamine maleate	34 (2)	27 (2.2)	7 (1.5)	0.889	0.346
Diphenhydramine	32 (1.9)	29 (2.3)	3 (0.6)	5.494	0.019*
Cimetidine	32 (1.9)	26 (2.1)	6 (1.3)	1.319	0.251
Mizolastine	31 (1.8)	21 (1.7)	10 (2.1)	0.319	0.572
System application glucocorticoids	26 (1.5)	23 (1.9)	3 (0.6)	3.462	0.063
Cyproheptadine	23 (1.3)	17 (1.4)	6 (1.3)	0.023	0.857
Montelukast sodium	11 (0.6)	9 (0.7)	2 (0.4)	0.506	0.477
Ranitidine	10 (0.6)	7 (0.6)	3 (0.6)	0.025	0.874
omalizumab	10 (0.6)	7 (0.6)	3 (0.6)	0.025	0.874
Promethazine	5 (0.3)	4 (0.3)	1 (0.2)	0.150	0.689

*p < 0.05; Chi-square test.

### Clinical Manifestations During Follow-Up

After 1 week of treatment, the proportion of severe itching dropped from 34.3 to 5.3%, and the proportion of patients with more than 50 wheals per 24 h dropped from 15.6 to 2.3%. In total, 1,715 outpatients with urticaria were examined for the first week follow-up, while 737 cases (43%) had abnormal laboratory values (with missing data in 18 cases). In total, 596 outpatients with urticaria completed follow-up once per week for a period of 1 month, and their urticaria activity score 7 (UAS7) showed a significant downward trend (week 0, just before the study, 27.44; week 1, 16.87; week 2, 12.39, week 3, 7.21; week 4, 4.2). The duration of the disease negatively correlated with the severity of itching and the number of wheals (>50/24H) (Spearman’s rank correlation test, *p* < 0.001).

## Discussion

This is an epidemiologic survey that focuses on clinical urticaria. However, comparing our results with previous studies is difficult, as the majority of those studies were population-based and relied on questionnaires (10). Given that only very limited epidemiologic information are available on the hospital-based study, features of clinical urticaria revealed by our study have provided valuable references that may be utilized for prevention, diagnosis, and management of urticaria in China.

The mean age of patients and the median duration of the disease were consistent with another study conducted in China (11). Most previous reports have demonstrated that women suffer from urticaria about two times moreoften than men do (12–15), based on highly selected patients from specialized urticaria treatment centers. In contrast, we identified a female-to-male ratio of 57:43 for urticaria and female dominance tend to increase with age in this hospital-based study (X^2^ = 9.194, *p*-value = 0.027, Pearson’s Chi-square test). The prevalence of CU in men might have been underestimated previously, as adult men may not be able to visit special clinics or participate in active studies in medical centers during regular working hours (15). This situation is not universal in China because the employment ratio of men to women has been aligned in cities. Thus, very little difference in the gender ratio of clinical urticaria is observed. In addition, a prominent theme among white middle-class men implicates “traditional masculine behavior” as an explanation for delays in seeking help among men who suffer from illness (16), resulting in an underestimated proportion of male urticaria.

In this hospital-based epidemiological survey, we estimated the proportion of each type of urticaria and angioedema for 1 year. The majority of patients suffer from chronic spontaneous urticaria (81.4%). The most common type of inducible urticaria is symptomatic dermographism (35.9%). This finding is consistent with previous studies (7–9). An investigation in Germany observed co-occurrence of angioedema with wheals in one-third of urticaria cases (33.3%). About 30–40% of those patients were diagnosed with isolated hive, whereas 10–20% with isolated angioedema, which is much higher than the individual proportion obtained in our survey (17). Almost all of those results were obtained from highly selected patients attending specialized urticaria treatment centers. Further comparisons with reports from other pieces of literature are difficult since definitions, numbers, and classification of urticaria subtypes vary considerably.

Few population-based studies have focused on epidemiology and comorbidities of chronic urticaria (CU). According to a nationwide population-based study in Korea, allergic rhinitis, drug allergies, asthma, thyroid diseases, and cancers were common comorbidities (18). Another study correlated CU with atopic/autoimmune diseases (19). Consistent with most observations, our data suggest a higher proportion of allergic diseases among patients with urticaria, including allergic rhinitis (18.8%), eczema/dermatitis (13.9%), and asthma (2.6%). A representative sample of 57,779 adults aged 20 years or older were recruited for the study by the national cross-sectional China Pulmonary Health (CPH), which showed that the overall prevalence of allergic rhinitis was 3.06% (20), which was much lower than our study. In respect of eczema and asthma, our results are similar to those reported by other studies (21).

As the third largest country in the world, there are huge climatic differences between northern and southern China, and in particular, the south has significantly high temperature and humidity. Perhaps the most interesting of all is that, as a multicenter cross-sectional epidemiological survey in different geographical locations in northern and southern China, this study filled the gap in the field of regional comparative studies on urticaria. There are several interesting things to notice here. First, more itching was observed in outpatients with urticaria from northern areas (99.5%), which is higher than that of the patients in the southern areas (94.1%, *P* < 0.001). We suspect that the dry skin caused by cold and dry weather in the northern areas is one of the major factors of itching. Second, the proportion of pain and arthralgia in northern areas is lower than that in southern areas (1.1 vs 5.1%, 0 vs 9.6%, *p* < 0.001). This is consistent with many studies (22) in China which have confirmed that the prevalence of joint diseases in the south is higher than that in the north. Third, in terms of family history, the specific mechanism for the big gap between the southern and the northern areas is unclear (10.6 vs 3.2%, *p* < 0.001). Perhaps familial heritability was related to ethnic aggregation, a larger sample of data is needed to support their correlation. In addition, there are currently limited studies on north–south differences in laboratory inspection; some studies suggest that there is no significant difference in the infection rate of *Helicobacter pylori* in chronic urticaria cases in different regions of the north and south China (23), which is not consistent with our findings. The relationship between *H. pylori* and urticaria seems to be unclear. The amount of total IgE was considered the simplest way to identify allergic subjects in the early studies. However, it became evident that total IgE levels could not be used as a reliable marker of allergy. In this survey, only 8.5% of patients who had been examined had an elevated serum total IgE level. Thus, low or normal values cannot exclude the presence of IgE-mediated diseases. As a consequence, total levels of IgE should be carefully interpreted and not considered as an indicator for the presence of allergic diseases (24). Our study indicates that the incidence of SD and cold urticaria in the south (68.5%) was lower than that in the north (76.8%), which is the extreme opposite of the previous studies (25), while the incidence of delayed pressure urticaria, heat urticaria, solar urticaria, in the south is higher than those in the north, and the difference is statistically significant. More interestingly, temperature-related urticaria seems to be consistent with geography and climate, and the cause of this association is not known, warranting further investigation.

Food allergens are defined as specific components of food or ingredients within food (typically proteins or chemical haptens) that can be recognized by allergen-specific immune cells to elicit specific immunologic reactions, resulting in characteristic symptoms (26). Cutaneous reaction to food is a common presentation of food allergy, including IgE-mediated (urticaria, angioedema, flushing, and pruritus), cell-mediated (contact dermatitis and dermatitis herpetiformis), and mixed IgE- and cell-mediated (atopic dermatitis) reactions (26). In this survey, half of the patients with urticaria have certain food items that need to be avoided; however, only 16.4% of them consider this approach as effective. Food allergy is rarely a cause of chronic urticaria (27), which is the major disease among the majority (81.4%) of the patient population in this study. Interestingly, fish–prawn–crab (30.7%) and alcohol consumption (20%) are the most popular food items to be avoided in China, in sharp contrast to nuts (1.8%) and beans (1%). This situation is quite different from other countries. The most prevalent studies have only focused on the most common foods, including egg, milk, peanut, tree nuts, wheat, and crustacean shellfish. The prevalence of peanut allergy in France, Germany, Israel, Sweden, and the United Kingdom varies between 0.06 and 5.9%. Moreover, the prevalence of seafood allergy in the United States was reported to be 5.9% (95% CI = 5.3–6.6%), where 2.3% (95% CI = 2–2.5%) was reported for any seafood allergy, 2% for shellfish, 0.4% for fish, and 0.2% for both types (28). Alcohol is considered to induce allergic reactions in China. Thus, there is a great difference of opinion over prohibited food items in different countries.

In terms of treatment, second-generation histamine H1-receptor antagonists (antihistamines) remain a front-line choice and are highly recommended by dermatologists, administered either separately or in combination. Due to limitations in medication guides, doubling dosage is rarely applied in practice. Overall, urticaria responded well to treatment, especially during the first week. Both the severity of itching or number of wheals were significantly reduced after 4°weeks of treatment. Interestingly, Ebastine was the preferred antihistamine prescribed by doctors. Most previous studies have demonstrated that Ebastine is generally well-tolerated. At therapeutic doses of 10 and 20°mg once every day, it had no adverse effects on cognitive functions and psychomotor performance or on cardiovascular functions (29). First-generation antihistamines, such as diphenhydramine and chlorphenamine (chlorpheniramine), provide effective relief of symptoms; however, they are very rarely used nowadays due to sedation and anticholinergic events (30).

Although glycyrrhizin is not an approved treatment for urticaria, up to one-third of the patients with urticaria, especially those patients with a disease course shorter than one year, received glycyrrhizin, which was more common than traditional Chinese medicine and even antihistamines. The oral compound glycyrrhizin is a preparation composed of glycyrrhizin, cysteine hydrochloride, and glycine. Its primary active component is glycyrrhizic acid extracted from licorice. Glycyrrhizic acid exhibits a remarkably broad spectrum of biological and pharmacological activities, including antitumor, anti-inflammatory, antioxidant, antiviral, antimicrobial, antiulcer, anti-diabetes, hepatoprotective, cardioprotective, and neuroprotective effects (17). The anti-inflammatory effects of glycyrrhizin were specific for membrane-dependent receptor-mediated stimuli. Although orally administered glycyrrhizin has been widely used in China for many years to treat urticaria, it has not been well reported due to a lack of clinical evidence. Compound glycyrrhizin in combination with conventional therapy enhances a clinical response. The use of compound glycyrrhizin should be formally expanded for this indication.

### Study Limitations and Strengths

This study has some limitations. First, because the participants were recruited from multiple centers, selection bias is inevitable due to a non-homogeneous population and spatial distribution. Second, routine laboratory tests, including autoantibody detection and the food allergy provocation test, were not performed for all patients due to limited conditions. Finally, the final course of the disease was not determined by long-term follow-up. Nevertheless, this study holds certain strengths. This hospital-based dermato-epidemiological study among Chinese outpatients includes a relatively large number of participants. This study presents epidemiologic features of clinical urticaria in different regions, based on diagnosis (ICD-10 codes) by dermatologists in real-world China. Although prevalence estimation is still exploratory and diagnosis of urticaria based on ICD-10 codes needs to be validated, this epidemiological study may help clinicians to educate patients with urticaria in addition to providing clues for future research endeavors.

## Conclusion

In conclusion, this study provides a profile of clinical urticaria in Chinese outpatients. CSU is the most common subtype among the participants. Allergic diseases are the most common concomitant disorders of urticaria. Fish–prawn–crab and alcohol consumption are often avoided by patients. Overall, clinical urticaria responds to treatment. Ebastine is the most frequently prescribed drug by doctors.

## Data Availability Statement

The raw data supporting the conclusions of this article will be made available by the authors, without undue reservation.

## Ethics Statement

The studies involving human participants were reviewed and approved by Urticaria Real World, 2019-P2-107-01, Beijing Friendship Hospital, Capital Medical University. Written informed consent from the participants or their legal guardian/next of kin was not required to participate in this study in accordance with the national legislation and the institutional requirements.

## Author Contributions

XW, L-JL, and L-FL performed the experiments and drafted the manuscript. X-DS performed the data analyses. Y-WS prepared the figures and tables. All authors reviewed the manuscript and approved the submitted version.

## Conflict of Interest

The authors declare that the research was conducted in the absence of any commercial or financial relationships that could be construed as a potential conflict of interest.

## Publisher’s Note

All claims expressed in this article are solely those of the authors and do not necessarily represent those of their affiliated organizations, or those of the publisher, the editors and the reviewers. Any product that may be evaluated in this article, or claim that may be made by its manufacturer, is not guaranteed or endorsed by the publisher.

## References

[1] Zuberbier T Aberer W Asero R Abdul Latiff AH Baker D Ballmer-Weber B The EAACI/GA2LEN/EDF/WAO guideline for the definition, classification, diagnosis and management of urticaria. *Allergy.* (2018) 73:1393–414. 10.1111/all.13397 29336054

[2] Zazzali JL Broder MS Chang E Chiu MW Hogan DJ. Cost, utilization, and patterns of medication use associated with chronic idiopathic urticaria. *Ann Allergy Asthma Immunol.* (2012) 108:98–102. 10.1016/j.anai.2011.10.018 22289728

[3] Lapi F Cassano N Pegoraro V Cataldo N Heiman F Cricelli I Epidemiology of chronic spontaneous urticaria: results from a nationwide, population-based study in Italy. *Br J Dermatol.* (2016) 174:996–1004. 10.1111/bjd.14470 26872037

[4] Pogorelov D Olisova O Nekrasova T Kolkhir P. Chronische spontane urtikaria in verbindung mit einer prim€ar billi€aren cholangitis: ein Fallbericht und Literaturu€berblick. *J Dtsch Dermatol Ges.* (2016) 14:1134–6. 27879068 10.1111/ddg.13064_g

[5] Xiao Y Huang X Jing D Huang Y Chen L Zhang X The prevalence of atopic dermatitis and chronic spontaneous Urticaria are associated with parental socioeconomic status in adolescents in China. *Acta Derm Venereol.* (2019) 99:321–6. 10.2340/00015555-3104 30521061

[6] Hagstrom EL Patel S Karimkhani C Boyers LN Williams HC Hay RJ Comparing cutaneous research funded by the US national institutes of health (NIH) with the US skin disease burden. *J Am Acad Dermatol.* (2015) 73:383–91. 10.1016/j.jaad.2015.04.039 26051697

[7] Magerl M Altrichter S Borzova E Giménez-Arnau A Grattan CE Lawlor F The definition, diagnostic testing, and management of chronic inducible urticarias – The EAACI/GA(2) LEN/EDF/UNEV consensus rec- ommendations 2016 update and revision. *Allergy.* (2016) 71:780–802. 10.1111/all.12884 26991006

[8] Weller K Schoepke N Krause K Ardelean E Bräutigam M Maurer M. Selected urticaria patients benefit from a referral to tertiary care centresdresults of an expert survey. *J Eur Acad Dermatol Venereol.* (2013) 27:e8–16. 10.1111/j.1468-3083.2011.04387.x 22176200

[9] Silpa-archa N Kulthanan K Pinkaew S. Physical urticaria: prevalence, type and natural course in a tropical country. *J Eur Acad Dermatol Venereol.* (2011) 25:1194–9. 10.1111/j.1468-3083.2010.03951.x 21175877

[10] Gaig P Olona M Munoz Lejarazu D Caballero MT Domínguez FJ Echechipia S Epidemiology of urticaria in Spain. *J Investig Allergol Clin Immunol.* (2004) 14:214–20. 15552715

[11] Zhong H Song Z Chen W Li H He L Gao T Chronic urticaria in Chinese population: a hospital-based multicenter epidemiological study. *Allergy.* (2014) 69:359–64. 10.1111/all.12338 24354882

[12] Zuberbier T Balke M Worm M Maurer M. Epidemiology of urticaria: a representative cross-sectional population survey. *Clin. Exp. Dermatol.* (2010) 35:869–73. 10.1111/j.1365-2230.2010.03840.x 20456386

[13] Lee HC Hong JB Chu CY. Chronic idiopathic urticaria in Taiwan: a clinical study of demographics, aggravating factors, laboratory findings, serum autoreactivity and treatment response. *J Formos Med Assoc.* (2011) 110:175–82. 21497281 10.1016/S0929-6646(11)60028-4

[14] Galdas PM Cheater F Marshall P. Men and health help-seeking behaviour: literature review. *J Adv Nurs.* (2005) 49:616–23. 10.1111/j.1365-2648.2004.03331.x 15737222

[15] Zuberbier T Balke M Worm M Edenharter G Maurer M. Epidemiology of urticaria: a representative cross-sectional population survey. *Clin Exp Dermatol.* (2010) 35:869–73. 20456386 10.1111/j.1365-2230.2010.03840.x

[16] Kim BR Yang S Choi JW Choi CW Youn SW. Epidemiology and comorbidities of patients with chronic urticaria in Korea: a nationwide population-based study. *J Dermatol.* (2018) 45:10–6. 10.1111/1346-8138.14075 28983950

[17] Chiu HY Muo CH Sung FC. Associations of chronic urticaria with atopic and autoimmune comorbidities: a nationwide population-based study. *Int J Dermatol.* (2018) 57:822–9. 10.1111/ijd.14000 29663342

[18] Huang K Yang T Xu J Yang L Zhao J Zhang X Prevalence, risk factors, and management of in China: a national cross-sectional study. *Lancet.* (2019) 394:407–18. 10.1016/S0140-6736(19)31147-X 31230828

[19] Rönmark EP Ekerljung L Mincheva R Sjölander S Hagstad S Wennergren G Different risk factor patterns for adult asthma, rhinitis and eczema: results from West Sweden Asthma Study. *Clin Transl Allergy.* (2016) 6:28–38. 10.1186/s13601-016-0112-0 27493721 PMC4973051

[20] Sun X Zhen X Hu X Li Y Gu S Gu Y Osteoarthritis in the middle-aged and elderly in China: prevalence and influencing factors. *Int J Environ Res Public Health.* (2019) 16:4701. 10.3390/ijerph16234701 31779104 PMC6926632

[21] Cui YL Zhou BY Gao GC. A systematic review and meta-analysis of the correlation between *Helicobacter pylori* infection and chronic urticaria. *Ann Palliat Med.* (2021) 10:10584–90. 34763505 10.21037/apm-21-2324

[22] Ansotegui IJ Melioli G Canonica GW Caraballo L Villa E Ebisawa M IgE allergy diagnostics and other relevant tests in allergy, a world allergy organization position paper. *World Allergy Organ J.* (2020) 13:100080. 32128023 10.1016/j.waojou.2019.100080 PMC7044795

[23] Breathnacht SM Allen R Ward AM Greaves MW. Symptomatic dermographism: natural history, clinical features, laboratory investigations and response to therapy. *Clin Exp Dermatol.* (1983) 8:463–76. 10.1111/j.1365-2230.1983.tb01814.x 6139191

[24] Niaid-Sponsored Expert Panel Boyce JA Assa’ad A Burks AW Jones SM Sampson HA Guidelines for the diagnosis and management of food allergy in the United States: report of the NIAID-sponsored expert panel. *J Allergy Clin Immunol.* (2010) 126:S1–58. 21134576 10.1016/j.jaci.2010.10.007 PMC4241964

[25] Burks W. Skin manifestations of food allergy. *Pediatrics.* (2003) 111:1617–24. 12777601

[26] Sicherer SH Munoz-Furlong A Sampson HA. Prevalence of seafood allergy in the United States determined by a random telephone survey. *J Allergy Clin Immunol.* (2004) 114:159–65. 10.1016/j.jaci.2004.04.018 15241360

[27] Sastre J. Ebastine in allergic rhinitis and chronic idiopathic urticaria. *Allergy.* (2008) 63:1–20. 10.1111/j.1398-9995.2008.01897.x 19032340

[28] Van Cauwenberge P Bachert C Passa- lacqua G Bousquet J Canonica GW Durham SR Consensus statement on the treatment of allergic rhinitis. European academy of allergology and clinical immunology. *Allergy.* (2000) 55:116–34. 10.1034/j.1398-9995.2000.00526.x 10726726

[29] van der Valk P Moret G Kiemeney L. The natural history of chronic urticaria and angioedema in patients visiting a tertiary referral centre. *Br J Dermatol.* (2002) 146:110–3. 10.1046/j.1365-2133.2002.04582.x 11841375

[30] Kulthanan K Jiamton S Thumpimukvatana N Pinkaew S. Chronic idiopathic urticaria: prevalence and clinical course. *J Dermatol.* (2007) 34:294–301. 10.1111/j.1346-8138.2007.00276.x 17408437

